# Differences in patterns of attention deficit/hyperactivity disorder medication use in US children

**DOI:** 10.1002/jcv2.70040

**Published:** 2025-09-25

**Authors:** Jennie E. Ryan, Alexander Weigard, Sean Esteban McCabe, Timothy E. Wilens, Philip T. Veliz

**Affiliations:** ^1^ College of Nursing Thomas Jefferson University Philadelphia Pennsylvania USA; ^2^ Department of Psychiatry University of Michigan Ann Arbor Michigan USA; ^3^ Center for the Study of Drugs, Alcohol, Smoking and Health and School of Nursing University of Michigan Ann Arbor Michigan USA; ^4^ Harvard University Medical School Cambridge Massachusetts USA

**Keywords:** attention deficit/hyperactivity disorder (ADHD), child and adolescent, health differences, pharmacotherapy, stimulant medication

## Abstract

**Background:**

Understanding attention deficit/hyperactivity disorder (ADHD) medication patterns is crucial for optimizing treatment outcomes. There are limited data on racial, ethnic, gender and socioeconomic treatment differences across longitudinal national samples.

**Methods:**

Secondary data analysis of baseline through 3rd‐year follow‐up (2016–2020) data from the Adolescent Brain Cognitive Development Study (ABCD) (*N* = 11,875). Data were collected from 21 US sites, to reflect national demographic diversity. Complete case panel included 9708 children aged 9/10 at baseline and 12/13 at 3rd‐year follow up. Sociodemographic factors (sex, race, ethnicity, household income), ADHD medication use (stimulants and non‐stimulants), and ADHD severity were examined.

**Results:**

By the 3rd‐year follow‐up, 13% of children used ADHD medications. Females were more likely to never have received medications compared to males (92% vs. 82%, odds ratio [OR] 2.34, 95% confidence interval [CI] 2.05–2.67, *p* < 0.001). Females exhibited lower mean ADHD severity scores, though the difference diminished over time. Asian (95% vs. 87%, OR 2.83, 95% CI 1.41–5.38, *p* < 0.001) and Hispanic children (90% vs. 86%, OR 1.39, 95% CI 1.17–1.64, *p* < 0.001) were more likely to have never received medications compared to White and non‐Hispanic children. Black children were more likely to discontinue medications (9% vs. 5%, OR 1.84, 95% CI 1.45–2.33, *p* < 0.001) compared to White children, although this was not significant after adjusting for sociodemographic factors. Children in lower income households were more likely to have clinically significant ADHD but less likely to receive and remain on medications compared to those from higher income households.

**Conclusions:**

Significant differences exist in ADHD medication use patterns among US children based on sex, race, ethnicity, and socioeconomic status. Addressing these differences is essential to ensure equitable access to treatment.

## Key Points

- What's known: Disparities in attention deficit/hyperactivity disorder (ADHD) medication use patterns have been observed across gender, race, ethnicity, and socioeconomic status. However, most prior studies rely on cross‐sectional data or medical records, which may underrepresent underserved populations.
- What's new: Using longitudinal data from the Adolescent Brain Cognitive Development Study (*N* = 9708) this study provides a nationally representative view of stimulant and non‐stimulant ADHD medication use from ages 9–13. Females, Asian, and Hispanic children were more likely to have never received ADHD medications, while Black children and those from lower‐income households were more likely to discontinue treatment over time.
- What's relevant: Findings emphasize the need for equitable, sustained access to ADHD treatment through culturally responsive care and policy reform.

## INTRODUCTION

The prevalence of attention deficit/hyperactivity disorder (ADHD) in the United States (US) has significantly increased in the past two decades (Xu et al., 2018) with an estimated 8.6% of children aged 5–11 years old, and 13.4% of children aged 12–17 years old, ever diagnosed with ADHD (Akinbami et al., 2011). ADHD is increasingly recognized as a disorder affecting individuals across the lifespan and is associated with multiple negative psychosocial outcomes (Klein et al., 2012) (including lower education/financial trajectories, unemployment, and poor marital outcomes) and negative health outcomes including mortality (Landes & London, 2021; London & Landes, 2016; Pelham et al., 2020). Medications commonly prescribed for ADHD, including stimulants and non‐stimulants, have been shown to reduce core ADHD symptoms (Wolraich et al., 2019) and mitigate long‐term negative outcomes (Arnold et al., 2015). However, only 62% of children with ADHD receive medications (Danielson, Bitsko, et al., 2018; Danielson, Visser, et al., 2018), and of those, over half discontinue medications (Biederman, Fried, et al., 2020; Biederman et al., 2019). Understanding patterns of ADHD medication use (adherence, nonadherence, discontinuation) is critical to optimize longterm benefits of pharmacotherapy for children with ADHD and inform clinical practice.

Significant disparities have been observed in ADHD pharmacotherapy based on race, ethnicity, sex, and socioeconomic status (SES). Prior research has shown that Black and Hispanic children are less likely to receive ADHD medications compared to White children (Zuvekas & Vitiello, 2012). Furthermore, Black and Hispanic families are more likely to discontinue medications compared to White non‐Hispanic families (Cummings et al., 2017; McCabe, 2002), though these disparities may be moderated by SES (Akinbami et al., 2011; Froehlich et al., 2007; Visser et al., 2014). Black children are more likely to receive behavioral therapy, while Hispanic children are less likely than White non‐Hispanic children to receive medication or behavioral therapy alone (Danielson, Bitsko, et al., 2018; Danielson, Visser, et al., 2018).

Historically, there has been a noted disparity in the diagnosis of ADHD between males and females, partly attributable to under‐recognition and/or referral bias in the assessment of female youth (Young et al., 2020). However, increased awareness has resulted in an upward trend in ADHD diagnosis and treatment for female children in the US (Fairman et al., 2020).

Research examining racial and ethnic disparities in ADHD diagnosis prevalence has been mixed. While the Diagnostic and Statistical Manual (DSM) of Mental Disorders, Fifth Edition, Text Revision (DSM‐5‐TR) reports lower ADHD diagnosis rates for Black and Hispanic children, and female children (American Psychiatric Association [APA], 2022), a recent meta‐analysis showed no significant difference in ADHD diagnosis by race and ethnicity (Cénat et al., 2022). Additionally, prior nationally representative longitudinal studies have highlighted sociodemographic disparities in ADHD diagnosis and treatment among US children (Morgan et al., 2013; Morgan & Hu, 2023), but these studies rely primarily on parent‐report of clinical ADHD diagnosis, which may be influenced by clinical biases and inconsistencies.

Several prior longitudinal studies have examined ADHD treatment disparities in diverse samples, some using direct behavioral measures (Morgan & Hu, 2023; Olfson et al., 2023). These studies have provided valuable insights into racial, ethnic, and socioeconomic disparities in ADHD diagnosis and medication use. However, many have relied on clinical diagnoses rather than standardized symptom rating scales or multi‐informant ADHD assessments, potentially overlooking children with significant ADHD symptomatology who lack a formal diagnosis. Additionally, few studies have comprehensively tracked ADHD medication patterns, including adherence, discontinuation, and treatment initiation, over multiple years while examining how these patterns intersect with sociodemographic factors.

While prior research has highlighted ADHD treatment disparities, additional work is needed to understand how ADHD symptom severity (rather than diagnosis alone) influences ADHD medication patterns over time. Furthermore, studies examining medication use often rely on medical records and insurance claims, which may exclude uninsured and underinsured populations, limiting the generalizability of findings (Adams et al., 2024; Costello et al., 2003; Danielson, Bitsko, et al., 2018; Danielson, Visser, et al., 2018; Froehlich et al., 2007; Kessler et al., 2012; Klein et al., 2012; Shi et al., 2021; Wilens et al., 2008).

To address this gap, we analyzed data from the Adolescent Brain Cognitive Development Study (ABCD), the largest (*N* = 11,875) longitudinal study of child and adolescent neurodevelopment and mental health in the US (Volkow et al., 2018). Although not nationally representative, the ABCD Study was designed to reflect the sociodemographic diversity of the US child population. A key strength of the ABCD dataset is the availability of validated behavioral assessments to examine subthreshold and full ADHD symptomatology across a diverse national sample. The primary aim of this study was to examine differences in ADHD medication patterns of use based on sex, race, ethnicity, and income. The secondary goal of this study was to assess if differences in ADHD symptom severity could explain disparities in ADHD medication use and non‐use.

## METHODS

### Data source

The study used baseline through 3rd year follow‐up data (2016–2020) from the ABCD Study, a large (*N* = 11,875) longitudinal study designed to assess brain and cognitive development from childhood (age 9/10 years old) through adolescence (Barch et al., 2018; Volkow et al., 2018). The study used a multistage probability sampling of public and private schools within 21 catchment sites to recruit children and parents to approximate the demographic and geographic diversity of the US child and adolescent population (Garavan et al., 2018). There was a collective recruitment effort to produce a baseline cohort who maximally reflected the diversity of the US child and adolescent population, which resulted in an oversampling of Black children and those living in rural areas (Barch et al., 2018; Garavan et al., 2018). Details regarding participant recruitment and sampling have been described in detail elsewhere (Barch et al., 2018; Garavan et al., 2018).

All children in sampled schools aged 9/10 years at baseline were invited to participate. Exclusion criteria included inability to understand or speak English or Spanish fluently, a contraindication to magnetic resonance image scanning, major medical or neurologic disorder, gestational age less than 28 weeks or birth weight less than 1200 g, current diagnosis of schizophrenia, moderate or severe autism spectrum disorder, history of traumatic brain injury, or unwillingness to complete assessments. The ABCD Study received centralized approval for data collection through the University of California Institutional Review Board (IRB), and at each individual catchment site. The study followed the Strengthening the Reporting of Observational Studies in Epidemiology reporting guidelines.

### Sociodemographic characteristics

Sociodemographic characteristics including sex assigned at birth, parent‐reported race and ethnicity, highest parental‐educational level, and combined household income were obtained from the PhenX toolkit (Barch et al., 2018). Race was determined based on parent response to the PhenX toolkit question “What race do you consider your child to be?” For purposes of analyses, parents who reported multiple races of the child were defined as “multi‐racial” and the subgroup “Asian” was defined as parent report of any of the following races; Asian Indian, Chinese, Filipino, Japanese, Korean, Vietnamese, and other Asian (see Supporting Information S1). The “Other” and “Unknown” race categories were excluded from tables due to heterogeneous composition and limited interpretability. Ethnicity was determined based on parent response to the PhenX toolkit question, “Do you consider the child Hispanic/Latino/Latina?” Youth whose parents answered “yes” were categorized as “Hispanic” and those who endorsed “no” were categorized as “Non‐Hispanic.” Race and ethnicity were modeled as distinct covariates in all analyses to reflect their conceptual and statistical independence.

### ADHD medication use

At each annual follow up, parents were asked what medications the child took in the past 2 weeks. Medications commonly employed for ADHD include stimulants and non‐stimulants, and parent‐report of those medications were determined to be for ADHD. All stimulant (amphetamine, dexmethylphenidate, dextroamphetamine, dextroamphetamine‐amphetamine, lisdexamfetamine, methamphetamine, and methylphenidate) and non‐stimulant (atomoxetine, clonidine, and guanfacine) medications were included in analyses. Please refer to Supporting Information S2 for specific details on how respondents were determined to be using ADHD medications (Bartsch et al., 2019; Weigard et al., 2023). For purposes of analyses, participants were categorized into four mutually exclusive patterns of ADHD medication use: (1) never use: no ADHD medication use reported at any wave; (2) discontinued use: ADHD medication use reported at baseline and not at later waves; (3) continuous use since 9/10 years of age: ADHD medication use reported at baseline and at each subsequent wave; and (4) continuous use after 9/10 years of age: no ADHD medication use reported at baseline (9/10 years old), but reported ADHD medication use at each subsequent wave. The term “continuous use” was used to denote reported use at each wave; however, because the ABCD Study assesses only past 2‐week use annually, this may not capture intermittent gaps or medication holidays between assessments. Medication data were available for 9708 youth with full medication histories, excluding 2170 cases due to attrition or missing data. A comparison table between participants in the complete case panel and those excluded from analyses due to missing medication data is presented in Appendix 1.

### ADHD symptom severity

Mental health symptoms and diagnoses were assessed using the Child Behavioral Checklist (CBCL) DSM five‐oriented scales (Achenbach & Ruffle, 2000; Biederman, DiSalvo, et al., 2020; Biederman, Fried, et al., 2020). The CBCL is a widely used and well‐validated parent‐reported inventory designed to assess emotional, behavioral, and social problems in children. It includes 113 items that caregivers rate based on their child's behavior over the past 6 months, providing standardized scores across various empirically derived and DSM‐oriented syndromes. In this study, we used the ADHD CBCL DSM‐5 Scale which generates age‐ and sex‐normed *t*‐scores (mean = 50, SD = 10), with scores >60 used in research to indicate clinically significant symptomatology (Biederman, DiSalvo, et al., 2020; Biederman, Fried, et al., 2020; Biederman et al., 1993; Chen et al., 1994; Spencer et al., 2018). The ADHD CBCL DSM‐5 Scale has been shown to correspond significantly with clinician‐rated ADHD diagnoses (Achenbach et al., 2001; Nakamura et al., 2009), with area under the curve values ranging from 0.69 to 0.87 depending on diagnostic subtype and comparison group (Ebesutani et al., 2010), supporting its proxy for clinically relevant ADHD symptoms. Details regarding reliability and validity of the CBCL, and its utilization in the ABCD Study, have been described elsewhere (Achenbach et al., 2001; Barch et al., 2018; Biederman, DiSalvo, et al., 2020; Biederman, Fried, et al., 2020; Biederman et al., 1993; Chen et al., 1994; Ebesutani et al., 2010; Miech et al., 2023; Nakamura et al., 2009; Spencer et al., 2018).

Because the ABCD dataset does not include clinical ADHD diagnoses, CBCL scores were used as a validated proxy for ADHD symptomatology to assess patterns of medication use. While stimulant and non‐stimulant medications are primarily prescribed for ADHD in pediatric populations, it is possible that some children received these medications for other conditions. However, given the established use of the CBCL ADHD‐oriented scale in epidemiological research, this approach provides a robust method for examining disparities in ADHD treatment.

### Statistical analyses

Statistical analyses were performed using STATA [StataCorp], version 18.0. All analyses accounted for clustering across the 21 research sites and within families using survey‐adjusted methods. The ABCD Study's recommended approach was followed to define clustered standard errors using site and family IDs, implemented via the “svyset” command in STATA with site and family specified as clustering variables. First, children were categorized based on ADHD medication use into four groups: never received medications, discontinued medication throughout the study period, continuous use since 9/10 years of age (i.e., used ADHD medication at baseline and continuously over the course of the study period), and continuous use after 9/10 years of age (i.e., started using ADHD medications continuously after baseline). Second, demographic characteristics of each group were evaluated. Unadjusted and adjusted logistic regression models were used to estimate odds ratios (ORs) and 95% confidence intervals (CIs) to assess differences between groups. Third, differences in stimulant and non‐stimulant medication use by sociodemographic characteristics (sex, race, ethnicity, household income, parental education) were assessed using a series of unadjusted logistic regressions across each wave of the study. Fourth, mean CBCL *t*‐scores were examined across each wave to assess ADHD symptom severity and a series of linear regression models were used to evaluate differences by sociodemographic characteristics. Finally, to assess ADHD symptom severity in relation to medication use, a series of logistic regression models were used to estimate unadjusted and adjusted odds ratios (AORs) with 95% CIs across each wave, stratified by sociodemographic variables (sex, race, ethnicity, and household income). All logistic regression models estimating AORs included sex, race, ethnicity, household income, and parental education as covariates. Where noted in the text, additional models further adjusted for ADHD symptom severity (CBCL *t*‐scores) to assess whether disparities in medication use patterns were explained by differences in symptom burden. These adjustments allowed for examination of the extent to which sociodemographic factors and ADHD severity independently contributed to observed disparities. The full set of model estimates and effect sizes to support interpretation of reported findings are found in text and the footnotes of all tables. While formal correction for multiple testing was not applied, analyses were structured around pre‐specified sociodemographic comparisons, and results were interpreted using effect sizes and CIs. A conservative significance threshold (*p* < 0.01) was used to reduce the likelihood of false positives. Missing data were handled using listwise deletion, ensuring that only complete cases were included in the analyses.

## RESULTS

### Patterns of use among full sample

By 3rd year follow up, 13% (*N* = 1278) of children had received an ADHD medication, 6% (*N* = 547) of children had discontinued ADHD medications, 4% (*N* = 428) of children had continuous use since 9/10 years of age (i.e., used ADHD medication at baseline and continuously over the course of the study period), and 3% (*N* = 303) of children had continuous use after 9/10 years of age (i.e., started using ADHD medications continuously after baseline) (Table 1). Among children receiving ADHD medications throughout the study period, 68% (*N* = 873) received a stimulant medication, 12% (*N* = 151) received a non‐stimulant medication, and 20% (*N* = 254) received both.

**TABLE 1:** Sociodemographic characteristics of children who never used, discontinued, or continuously used attention deficit/hyperactivity disorder medications.

	Never use			Discontinued			Continuous use after 9/10 yo			Continuous use since 9/10 yo		
	*N* (%)^a^	OR (95% CI)	aOR^b^ (95% CI)	*N* (%)^a^	OR (95% CI)	aOR^b^ (95% CI)	*N* (%)^a^	OR (95% CI)	aOR^b^ (95% CI)	*N* (%)^a^	OR (95% CI)	aOR^b^ (95% CI)
Total	8430 (87)			547 (6)			303 (3)			428 (4)		
Race												
White	5577 (87)	Ref	Ref	328 (5)	Ref	Ref	218 (3)	Ref	Ref	315 (5)	Ref	Ref
Black/African American	1121 (86)	0.96 (0.49–1.15)	1.14 (0.90–1.45)	117 (9)	1.84** (1.45–2.33)	1.33 (0.96–1.83)	23 (2)	0.51* (0.33–0.79)	0.54 (0.32–0.91)	41 (3)	0.63* (0.44–0.91)	0.60 (0.37–0.95)
American Indian/Alaska Native	41 (87)	1.06 (0.44–2.51)	1.00 (0.39–2.59)	5 (11)	2.22 (0.87–5.68)	1.77 (0.61–5.15)	1 (2)	0.62 (0.09–4.52)	0.87 (0.12–6.48)	0	NA	NA
Asian	183 (95)	2.83* (1.41–5.68)	2.82* (1.32–6.02)	3 (2)	0.29 (0.09–0.92)	0.23 (0.6–0.95)	5 (3)	0.76 (0.31–1.86)	0.83 (0.34–2.04)	2 (1)	0.20 (0.05–0.82)	0.22 (0.50–0.88)
Native Hawaiian/Other Pacific Islander	12 (100)	NA	NA	0	NA	NA	0	NA	NA	0	NA	NA
Multiracial	1050 (86)	0.93 (0.77–1.12)	0.96 (0.78–1.17)	72 (6)	1.16 (0.89–1.53)	1.02 (0.75–1.38)	46 (4)	1.11 (0.81–1.54)	1.21 (0.46–1.05)	57 (5)	0.95 (0.70–1.29)	0.96 (0.69–1.34)
Sex assigned at birth												
Male	4197 (82)	Ref	Ref	377 (7)	Ref	Ref	194 (4)	Ref	Ref	321 (6)	Ref	Ref
Female	4231 (92)	2.34** (2.05–2.67)	2.25** (1.97, −2.59)	170 (4)	0.48** (0.39–0.58)	0.52** (0.42–0.63)	108 (2)	0.60** (0.47–0.76)	0.64** (0.50–0.82)	107 (2)	0.35** (0.28–0.44)	0.33** (0.26–0.42)
Ethnicity												
Non‐Hispanic	6649 (86)	Ref	Ref	433 (6)	Ref	Ref	256 (3)	Ref	Ref	373 (5)	Ref	Ref
Hispanic	1688 (90)	1.39** (1.17–1.64)	1.40* (1.14–1.73)	104 (6)	0.98 (0.78–1.24)	0.95 (0.72–1.26)	41 (2)	0.65 (0.46–0.91)	0.69 (0.46–1.03)	49 (3)	0.53** (0.39–0.72)	0.54* (0.36–0.80)
Household income												
Below poverty line (<$24 k)	810 (85)	0.83 (0.67–1.02)	0.66* (0.50–0.88)	81 (9)	2.22** (1.67–2.95)	2.00** (1.37–2.92)	28 (3)	0.81 (0.53–1.24)	1.10 (0.63–1.92)	29 (3)	0.64 (0.44–0.98)	1.15 (0.67–2.00)
$25,000–$49,000	875 (85)	0.79 (0.64–0.97)	0.70* (0.55–0.89)	92 (9)	2.32** (1.77–3.04)	2.11** (1.52–2.93)	21 (2)	0.55* (0.35–0.88)	0.69 (0.42–1.15)	43 (4)	0.88 (0.60–1.29)	1.19 (0.77–1.83)
$50,000–$100,000	2060 (87)	0.98 (0.83–1.14)	0.95 (0.80–1.12)	128 (5)	1.36* (1.07–1.73)	1.29 (1.00–1.65)	67 (3)	0.78 (0.58–1.04)	0.89 (0.66–1.19)	103 (4)	0.92 (0.72–1.19)	0.98 (0.75–1.29)
>$100,000	4019 (88)	Ref	Ref	186 (4)	Ref	Ref	166 (4)	Ref	Ref	216 (5)	Ref	Ref
Parent education												
Less than high school	320 (88)	1.07 (0.77–1.5)	1.49 (0.92–2.44)	24 (7)	1.48 (0.95–2.32)	0.59 (0.30–1.14)	9 (3)	0.71 (0.36–1.40)	1.22 (0.55–2.69)	10 (3)	0.59 (0.29–1.19)	0.594 (0.16–1.82)
High school	883 (86)	0.90 (0.73–1.11)	0.99 (0.75–1.30)	91 (9)	2.04** (1.56–2.66)	1.30 (0.91–1.84)	25 (2)	0.70 (0.46–1.06)	0.97 (0.57–1.65)	26 (3)	0.54* (0.35–0.84)	0.60 (0.34–1.05)
Some college	1207 (86)	0.88 (0.74–1.06)	0.93 (0.75–1.14)	83 (6)	1.31 (1.00–1.72)	1.01 (0.75–1.37)	45 (3)	0.92 (0.66–1.28)	1.05 (0.72–1.53)	69 (5)	1.08 (0.80–1.45)	1.18 (0.84–1.66)
Associate's degree	1063 (85)	0.84 (0.70–1.00)	0.92 (0.75–1.14)	89 (7)	1.61** (1.24–2.08)	1.23 (0.92–1.67)	29 (2)	0.66 (0.44–0.99)	0.79 (0.51–1.21)	65 (5)	1.15 (0.85–1.54)	1.09 (0.78–1.53)
Bachelor's degree or higher	4920 (87)	Ref	Ref	257 (5)	Ref	Ref	195 (4)	Ref	Ref	258 (5)	Ref	Ref

*Note:* Bold indicates statistical significance. *Indicates *p* value ≤ 0.010; **indicates *p* value ≤ 0.001.

Abbreviations: aOR, adjusted odds ratio; CI, confidence interval; NA, not applicable; OR, odds ratio; ref, reference group.

^a^ Percentages are weighted.

^b^ All aOR models are adjusted for sociodemographic including race, sex, ethnicity, household income and parent education as reported in follow up year 3.

### Patterns of use by sex

Compared to male children, female children were more likely to have never received ADHD medications (92% [*N* = 4231] vs. 82% [*N* = 4197], OR 2.34 [95% CI 2.05–2.67], *p* < 0.001), less likely to discontinue ADHD medications (4% [*N* = 170] vs. 7% [*N* = 377], OR 0.48 [95% CI 0.39–0.58], *p* < 0.001), less likely to have continuous use after 9/10 years of age (2% [*N* = 108] vs. 4% [*N* = 194], OR 0.60 [95% CI 0.47–0.76], *p* < 0.001), and less likely to have continuous use since 9/10 years of age (2% [*N* = 107] vs. 6% [*N* = 321], OR 0.35 [95% CI 0.28–0.44], *p* < 0.001) (Table 1). Across each wave of follow‐up data, females were less likely to receive both stimulant and non‐stimulant medications (Table 2). These findings remained statistically significant after adjusting for race, ethnicity, parental education, and household income.

**TABLE 2:** Attention deficit/hyperactivity disorder medication use across each wave of the study period, stratified by sociodemographic characteristics.

	Baseline stimulant			Baseline non‐stimulant			Year 1 stimulant			Year 1 non‐stimulant		
	*N* (%)^a^	OR (95% CI)	aOR^b^ (95% CI)	*N* (%)^a^	OR (95% CI)	aOR^b^ (95% CI)	*N* (%)^a^	OR (95% CI)	aOR^b^ (95% CI)	*N* (%)^a^	OR (95% CI)	aOR^b^ (95% CI)
Total	913 (8)			284 (3)			865 (7)			287 (3)		
Race												
White	591 (8)	Ref	Ref	188 (3)	Ref	Ref	587 (8)	Ref	Ref	191 (3)	Ref	Ref
Black/African American	158 (8)	1.08 (0.90–1.30)	0.85 (0.66–1.10)	50 (3)	1.07 (0.78–1.47)	0.63 (0.40–0.98)	200 (7)	0.96 (0.78–1.17)	0.81 (0.61–1.07)	43 (3)	0.97 (0.70–1.36)	0.64 (0.38–1.07)
American Indian/Alaska Native	3 (5)	0.59 (0.19–1.90)	0.60 (0.18–2.05)	0	NA	NA	3 (5)	0.63 (0.20–2.01)	0.49 (0.12–2.06)	1 (2)	0.66 (0.09–4.77)	0.66 (0.08–5.17)
Asian	6 (2)	0.30* (0.13–0.67)	**0.30 (0.12–0.75)**	**0**	NA	NA	5 (2)	0.25* (0.10–0.61)	0.30 (0.10–0.85)	1 (<1)	0.16 (0.02–1.15)	0.19 (0.03–1.39)
Native Hawaiian/Other Pacific Islander	0 (0)	NA	NA	0	NA	NA	0	NA	NA	0	NA	NA
Multiracial	124 (8)	1.05 (0.86–1.29)	1.02 (0.81–1.28)	37 (2)	0.98 (0.69–1.4)	0.94 (0.64–1.38)	114 (8)	1.00 (0.81–1.23)	0.95 (0.75–1.20)	43 (3)	1.16 (0.83–1.62)	1.12 (0.78–1.62)
Sex assigned at birth												
Male	670 (11)	Ref	Ref	216 (3)	Ref	Ref	614 (10)	Ref	Ref	218 (4)	Ref	Ref
Female	243 (4)	0.37** (0.32–0.43)	0.38** (0.32–0.45)	68 (1)	0.34** (0.25–0.44)	0.35** (0.26–0.47)	251 (5)	0.42** (0.36–0.49)	0.43** (0.36–0.51)	69 (1)	0.34** (0.26–0.44)	0.34** (0.26–0.46)
Ethnicity												
Non‐Hispanic	751 (8)	Ref	Ref	245 (3)	Ref	Ref	715 (8)	Ref	Ref	247 (3)	Ref	Ref
Hispanic	154 (6)	0.78* (0.65–0.93)	0.77 (0.61–0.98)	35 (1)	0.55** (0.38–0.78)	0.42** (0.26–0.67)	137 (6)	0.75* (0.62–0.91)	0.81 (0.63–1.04)	34 (2)	0.54** (0.38–0.78)	0.41** (0.26–0.65)
Household income												
Below poverty line ($24 k)	163 (10)	1.52** (1.25–1.85)	1.56* (1.18–2.08)	55 (3)	2.08** (1.46–2.96)	2.60** (1.61–4.20)	123 (9)	1.31 (1.06–1.63)	1.62* (1.19–2.18)	59 (4)	2.39** (1.71–3.34)	3.45** (2.07–5.76)
$25,000–$49,000	142 (9)	1.35* (1.1–1.66)	1.32 (1.01–1.72)	61 (4)	2.39** (1.7–3.37)	2.69** (1.74–4.15)	114 (8)	1.15 (0.92–1.43)	1.25 (0.95–1.65)	47 (3)	1.79* (1.25–2.57)	2.10* (1.32–3.34)
$50,000–$100,000	227 (7)	1.10 (0.91–1.32)	1.03 (0.84–1.25)	75 (2)	1.5 (1.08–2.07)	1.45 (1.02–2.07)	225 (8)	1.16 (0.97–1.38)	1.16 (0.95–1.41)	76 (3)	1.47 (1.08–2.01)	1.60* (1.13–2.24)
>$100,000	310 (7)	Ref	Ref	75 (2)	Ref	Ref	329 (7)	Ref	Ref	87 (2)	Ref	Ref
Parent education												
Less than high school	37 (6)	0.94 (0.66–1.33)	0.87 (0.53–1.42)	12 (2)	1.10 (0.60–2.00)	1.01 (0.46–2.19)	29 (6)	0.80 (0.54–1.18)	0.69 (0.42–1.15)	16 (3)	1.53 (0.90–2.60)	1.18 (0.60–2.36)
High school	120 (8)	1.31 (1.06–1.61)	1.20 (0.90–1.61)	37 (3)	1.42 (0.98–2.07)	1.23 (0.76–1.98)	89 (7)	0.98 (0.78–1.24)	0.96 (0.68–1.34)	36 (3)	1.36 (0.93–1.97)	0.99 (0.59–1.66)
Some college	190 (10)	1.56** (1.3–1.86)	1.36* (1.08–1.71)	61 (3)	1.75** (1.27–2.39)	1.34 (0.91–1.98)	162 (9)	1.24 (1.03–1.5)	1.16 (0.92–1.47)	48 (3)	1.23 (0.88–1.72	0.96 (0.64–1.43)
Associate's degree	154 (10)	1.60** (1.32–1.95)	1.52** (1.21–1.91)	59 (4)	2.16** (1.57–2.97)	1.79* (1.24–2.59)	139 (9)	1.30 (1.06–1.59)	1.18 (0.93–1.50)	55 (4)	1.73** (1.26–2.38)	1.33 (0.91–1.93)
Bachelor's degree or higher	411 (7)	Ref	Ref	115 (2)	Ref	Ref	444 (7)	Ref	Ref	131 (2)	Ref	Ref

	Year 2 stimulant			Year 2 non‐stimulant			Year 3 stimulant			Year 3 non‐stimulant		
	*N* (%)^a^	OR (95% CI)	aOR^b^ (95% CI)	*N* (%)^a^	OR (95% CI)	aOR^b^ (95% CI)	*N* (%)^a^	OR (95% CI)	aOR^b^ (95% CI)	*N* (%)^a^	OR (95% CI)	aOR^b^ (95% CI)
Total *N* (%)^a^	882 (8)			288 (3)			763 (8)			243 (2)		
Race												
White	618 (9)	Ref	Ref	204 (3)	Ref	Ref	543 (8)	Ref	Ref	177 (3)	Ref	Ref
Black/African American	116 (7)	0.80 (0.65–0.98)	0.71 (0.54–0.93)	33 (2)	0.69 (0.48–1.01)	0.48* (0.28–0.81)	77 (6)	0.66** (0.51–0.84)	0.66* (0.49–0.91)	24 (2)	0.64 (0.42–0.98)	0.46 (0.24–0.86)
American Indian/Alaska Native	1 (2)	0.20 (0.03–1.47)	0.25 (0.3–1.82)	1 (2)	0.66 (0.09–4.77)	0.84 (0.12–5.74)	2 (4)	0.47 (0.11–1.93)	0.70 (0.16–2.99)	0	NA	NA
Asian	5 (3)	0.24* (0.10–0.59)	0.27 (0.09–0.77)	0	NA	NA	7 (3)	0.40 (0.19–0.86)	0.44 (0.18–1.04)	0	NA	NA
Native Hawaiian/Other Pacific Islander	0	NA	NA	0	NA	NA	0	NA	NA	0	NA	NA
Multiracial	117 (9)	0.96 (0.78–1.18)	0.95 (0.76–1.20)	40 (3)	1.00 (0.71–1.41)	1.00 (0.69–1.46)	108 (8)	1.03 (0.83–1.28)	1.05 (0.82–1.34)	34 (3)	0.99 (0.68–1.44)	0.95 (0.64–1.42)
Sex assigned at birth												
Male	623 (11)	Ref	Ref	215 (4)	Ref	Ref	539 (10)	Ref	Ref	185 (3)	Ref	Ref
Female	259 (5)	0.43** (0.37–0.50)	0.44** (0.38–0.52)	73 (1)	0.37** (0.28–0.48)	0.39** (0.29–0.52)	224 (5)	0.44** (0.37–0.51)	0.45** (0.38–0.53)	58 (1)	0.34** (0.25–0.46)	0.34** (0.25–0.47)
Ethnicity												
Non‐Hispanic	741 (9)	Ref	Ref	251 (3)	Ref	Ref	646 (8)	Ref	Ref	215 (3)	Ref	Ref
Hispanic	131 (6)	0.69** (0.57–0.84)	0.74 (0.56–0.95)	32 (2)	0.51** (0.34–0.74)	0.29** (0.17–0.51)	105 (5)	0.62** (0.50–0.77)	0.65* (0.49–0.86)	24 (1)	0.44** (0.28–0.67)	0.36** (0.20–0.62)
Household income												
Below poverty line ($24 k)	101 (8)	1.01 (0.80–1.27)	1.33 (0.97–1.83)	44 (4)	1.67* (1.17–2.38)	2.99** (1.84–4.85)	72 (7)	0.82 (0.63–1.07)	1.20 (0.84–1.71)	28 (3)	1.28 (0.84–1.95)	3.00** (1.68–5.37)
$25,000–$49,000	96 (7)	1.05 (0.84–1.31)	1.31 (0.99–1.72)	35 (3)	1.22 (0.83–1.8)	1.79 (1.14–2.81)	65 (6)	0.07 (0.53–0.92)	0.88 (0.63–1.23)	30 (3)	1.30 (0.86–1.97)	2.10* (1.30–3.41)
$50,000–$100,000	227 (8)	1.03 (0.87–1.22)	1.08 (0.89–1.31)	70 (3)	1.16 (0.85–1.57)	1.28 (0.92–1.78)	169 (8)	0.82 (0.68–0.99)	0.89 (0.73–1.09)	60 (3)	1.16 (0.84–1.61)	1.38 (0.98–1.94)
>$100,000	377 (8)	Ref	Ref	109 (2)	Ref	Ref	392 (8)	Ref	Ref	100 (2)	Ref	Ref
Parent education												
Less than high school	26 (6)	0.71 (0.48–1.05)	0.75 (0.45–1.25)	15 (3)	1.35 (0.78–2.31)	0.99 (0.46–2.13)	25 (6)	0.76 (0.50–1.15)	0.92 (0.51–1.67)	5 (1)	0.63 (0.28–1.43)	0.50 (0.16–1.60)
High school	83 (7)	0.91 (0.72–1.15)	0.99 (0.72–1.36)	28 (23)	0.94 (0.62–1.41)	0.69 (0.38–1.21)	59 (5)	0.65* (0.49–0.85)	0.72 (0.49–1.06)	18 (2)	0.69 (0.42–1.13)	0.53 (0.27–1.05)
Some college	148 (9)	1.11 (0.92–1.35)	1.16 (0.92–1.46)	47 (3)	1.19 (0.85–1.67)	1.10 (0.75–1.60)	116 (8)	0.98 (0.79–1.21)	1.10 (0.85–1.42)	44 (3)	1.28 (0.91–1.80)	1.00 (0.65–1.52)
Associate's degree	133 (9)	1.11 (0.90–1.36)	1.16 (0.92–1.47)	56 (4)	1.71* (1.25–2.35)	1.48 (1.00–2.19)	96 (7)	0.92 (0.73–1.15)	0.96 (0.73–1.25)	39 (3)	1.29 (0.9–1.85)	1.02 (0.66–1.58)
Bachelor's degree or higher	469 (8)	Ref	Ref	141 (2)	Ref	Ref	465 (8)	Ref	Ref	136 (2)	Ref	Ref

*Note*: Bold indicates statistical significance. *Indicates *p* value ≤ 0.010; **indicates *p* value ≤ 0.001.

Abbreviations: aOR, adjusted odds ratio; CI, confidence interval; NA, not applicable; OR, odds ratio; ref, reference group.

^a^ Percentages are weighted.

^b^ All aOR models were adjusted for sociodemographic factors including race, sex, ethnicity, household income, and parent education as reported in corresponding study wave.

At baseline and each wave of follow‐up, female children had statistically significant lower mean CBCL *t*‐scores (Table 3). As presented in Table 4, females were less likely to have elevated CBCL *t*‐scores at baseline and year 1, but more likely to have elevated CBCL *t*‐scores at year 3 compared to males (13% [*N* = 622] vs. 11% [*N* = 602], OR 1.17 (95% CI 1.04–1.33, *p* < 0.01)). By years 2 and 3, females were more likely to have elevated CBCL *t*‐scores and not receive ADHD medications compared to males (Table 4). These findings remained statistically significant after adjusting for race, ethnicity, parental education, and household income.

**TABLE 3:** Mean Child Behavioral Checklist *t*‐scores across each wave of the study period stratified by sociodemographic characteristics.

	CBCL score	95% CI	CBCL score	95% CI	CBCL score	95% CI	CBCL score	95% CI
	Baseline		Year 1 mean (SD)		Year 2 mean (SD)		Year 3 mean (SD)	
	Mean (SD)							
Sex								
Male	53.66 (6.02)	Ref	53.35 (5.77)	Ref	53.32 (5.53)	Ref	53.49 (5.56)	Ref
Female	52.76 (5.14)	**[−1.11, −0.71]**	52.59 (4.95)	**[−0.96, −0.55]**	52.88 (5.12)	**[−0.64, −0.23]**	53.29 (5.27)	**[−0.41, −0.02]**
Household income								
Below poverty line ($24 k)	54.38 (6.54)	**[1.46, 2.19]**	53.91 (6.27)	**[1.17, 1.90]**	53.72 (5.79)	**[0.64, 1.37]**	53.73 (5.58)	[0.23, 0.99]
$250,000–$49,000	53.83 (6.08)	**[0.94, 1.63]**	53.65 (5.88)	**[0.93, 1.62]**	53.56 (5.70)	**[0.48, 1.20]**	53.97 (5.76)	**[0.45, 1.24]**
$50,000–$100,000	53.23 (5.65)	**[0.42, 0.03]**	53.15 (5.57)	**[0.53, 1.03]**	53.25 (5.50)	**[0.28, 0.79]**	53.63 (5.61)	**[0.24, 0.79]**
>$100,000	52.55 (4.93)	Ref	52.37 (4.69)	Ref	52.72 (4.90)	Ref	53.12 (5.20)	Ref
Ethnicity								
Non‐Hispanic	53.24 (5.66)	Ref	53.03 (5.47)	Ref	53.15 (5.37)	Ref	53.45 (5.49)	Ref
Hispanic	53.18 (5.52)	[−0.31, 0.20]	52.84 (6.17)	[−0.43, 0.06]	52.98 (5.18)	[−0.42, 0.08]	53.18 (5.15)	[−0.52, −0.01]
Race								
White	53.00 (5.41)	Ref	52.86 (5.31)	Ref	53.03 (5.25)	Ref	53.34 (5.38)	Ref
Black/African American	53.88 (6.21)	**[0.56, 1.20]**	53.43 (5.78)	**[0.25, 0.88]**	53.24 (5.2)	[−0.10, 0.52]	53.47 (5.53)	[−0.20, −0.46]
American Indian/Alaska Native	53.34 (6.71)	[−1.34, 2.02]	53.63 (6.45)	[−0.91, 2.45]	54.52 (7.57)	[−0.54, 3.51]	54.10 (6.63)	[−1.11, 2.62]
Asian	51.52 (3.48)	**[−1.93, −1.02]**	51.36 (3.33)	**[−1.95, −1.05]**	51.68 (3.33)	**[−1.82, −0.90]**	51.56 (3.43)	**[−2.28, −1.29]**
Native Hawaiian/Other Pacific Islander	54.13 (7.01)	[−2.31, 4.58]	51.93 (2.87)	[−2.51, 0.64]	51.36 (3.46)	[−3.53, 0.17]	52.75 (4.88)	[−3.01, 1.82]
Multiracial	53.87 (6.21)	**[0.53, 1.23]**	53.53 (5.87)	**[0.33, 1.01]**	53.69 (5.82)	**[0.31, 1.01]**	54.06 (5.77)	**[0.36, 1.07]**

*Note*: Bold indicates *p* value ≤ 0.001.

Abbreviations: CBCL, Child Behavioral Checklist; CI, confidence interval; SD, standard deviation.

**TABLE 4:** Multivariable results: CBCL *t*‐scores, ADHD medication use and non‐use, stratified by sex, race, ethnicity, and socioeconomic status.

	Baseline			Year 1			Year 2			Year 3		
	*N* (%)^a^	OR (95% CI)	aOR^b^ (95% CI)	*N* (%)^a^	OR (95% CI)	aOR^b^ (95% CI)	*N* (%)^a^	OR (95% CI)	aOR^b^ (95% CI)	*N* (%)^a^	OR (95% CI)	aOR^b^ (95% CI)
CBCL *t*‐score >60												
Sex												
Male	912 (15)	Ref	Ref	772 (13)	Ref	Ref	602 (11)	Ref	Ref	602 (11)	Ref	Ref
Female	666 (12)	0.77** (0.69–0.86)	0.75** (0.58–0.85)	579 (11)	0.80** (0.71–0.90)	0.80** (0.71–0.91)	622 (13)	0.95 (0.84–1.06)	0.98 (0.86–1.11)	622 (13)	1.17* (1.04–1.32)	1.20* (1.06–1.37)
Race												
Black	319 (17)	1.49** (1.30–1.73)	1.05* (0.87–1.27)	247 (15)	1.35** (0.60–2.68)	0.95 (0.78–1.17)	200 (12)	1.08 (0.91–1.28)	0.87 (0.70–1.08)	186 (13)	1.17 (0.98–1.39)	0.97 (0.78–1.21)
Other	1261 (13)	Ref	Ref	1106 (12)	Ref	Ref	1076 (12)	Ref	Ref	1038 (12)	Ref	Ref
Asian	11 (5)	0.34* (0.16–0.68)	0.35* (0.18–0.69)	9 (4)	0.32** (0.16–0.62)	0.33* (0.16–0.68)	10 (5)	0.37* (0.19–0.69)	0.44 (0.23–0.84)	9 (4)	0.36* (0.18–0.69)	0.31* (0.15–0.66)
Other	1569 (14)	Ref	Ref	1344 (12)	Ref	Ref	1266 (12)	Ref	Ref	1215 (12)	Ref	Ref
Ethnicity												
Non‐Hispanic	1234 (13)	Ref	Ref	1085 (12)	Ref	Ref	1024 (12)	Ref	Ref	986 (12)	Ref	Ref
Hispanic	324 (14)	1.02 (0.89–1.16)	0.91 (0.78–1.07)	248 (11)	0.90 (0.78–1.04)	0.80 (0.67–0.95)	235 (11)	0.91 (0.79–1.06)	0.80 (0.66–0.96)	222 (11)	0.88 (0.75–1.02)	0.83 (0.69–1.00)
SES												
<$100 K	968 (15)	1.62** (1.43–1.83)	1.34** (1.16–1.55)	814 (14)	1.69** (1.49–1.93)	1.62** (1.39–1.87)	682 (13)	1.41** (1.24–1.61)	1.38** (1.18–1.60)	620 (14)	1.29** (1.14–1.47)	1.29** (1.12–1.5)
>$100 K	461 (10)	Ref	Ref	427 (9)	Ref	Ref	469 (10)	Ref	Ref	510 (11)	Ref	Ref
No ADHD medication and CBCL *t*‐score >60												
Sex												
Male	546 (9)	Ref	Ref	444 (8)	Ref	Ref	382 (7)	Ref	Ref	363 (7)	Ref	Ref
Female	511 (9)	1.02 (0.90–1.16)	1.01 (0.88–1.15)	438 (8)	1.09 (0.95–1.25)	1.08 (0.94–1.26)	446 (9)	1.32** (1.14–1.52)	1.33** (1.15–1.56)	486 (10)	1.54** (1.34–1.78)	1.58** (1.36–1.83)
Race												
Black	234 (13)	1.75** (1.48–2.05)	1.24 (1.02–1.53)	173 (10)	1.54** (1.28–1.85)	1.05 (0.84–1.32)	143 (9)	1.28 (1.05–1.57)	1.03 (0.80–1.31)	151 (11)	1.46** (1.20–1.77)	1.13 (0.89–1.44)
Other	825 (8)	Ref	Ref	711 (7)	Ref	Ref	685 (7)	Ref	Ref	698 (8)	Ref	Ref
Asian	10 (4)	0.52 (0.28–0.99)	0.60 (0.31–1.17)	9 (4)	0.54 (0.28–1.06)	0.57 (0.28–1.18)	10 (5)	0.64 (0.33–1.20)	0.80 (0.42–1.54)	7 (3)	0.43 (0.10–0.92)	0.36 (0.15–0.88)
Other	1049 (9)	Ref	Ref	875 (8)	Ref	Ref	818 (8)	Ref	Ref	842 (9)	Ref	Ref
Ethnicity												
Non‐Hispanic	791 (9)	Ref	Ref	686 (8)	Ref	Ref	636 (7)	Ref	Ref	661 (8)	Ref	Ref
Hispanic	249 (10)	1.24* (1.07–1.44)	1.13 (0.94–1.38)	183 (8)	1.07 (0.90–1.27)	0.94 (0.76–1.15)	181 (8)	1.16 (0.97–1.37)	1.02 (0.82–1.26)	175 (9)	1.05 (0.88–1.25)	0.99 (0.80–1.22)
SES												
<$100 K	655 (10)	1.69** (1.46–1.96)	1.29* (1.09–1.55)	534 (9)	1.74** (1.49–2.03)	1.54** (1.28–1.85)	456 (9)	1.57** (1.34–1.84)	1.38** (1.14–1.67)	463 (10)	1.54** (1.32–1.79)	1.40** (1.18–1.68)
>$100 K	293 (6)	Ref	Ref	268 (6)	Ref	Ref	280 (6)	Ref	Ref	322 (7)	Ref	Ref
ADHD medication and CBCL *t*‐score >60												
Sex												
Male	366 (6)	Ref	Ref	328 (6)	Ref	Ref	303 (5)	Ref	Ref	239 (5)	Ref	Ref
Female	155 (3)	0.45** (0.37–0.54)	0.46** (0.37–0.56)	141 (3)	0.46** (0.37–0.56)	0.47** (0.39–0.58)	145 (3)	0.51** (0.42–0.63)	0.56** (0.45–0.69)	136 (3)	0.62** (0.50–0.77)	0.65** (0.52–0.82)
Race												
Black	85 (5)	1.01 (0.78–1.31)	0.76 (0.53–1.08)	57 (4)	0.77 (0.57–1.05)	0.81 (0.56–1.18)	94 (4)	1.05 (0.83–1.32)	0.95 (0.72–1.26)	35 (3)	0.63 (0.43–0.91)	0.98 (0.71–1.34)
Other	436 (4)	Ref	Ref	391 (4)	Ref	Ref	353 (4)	Ref	Ref	340 (4)	Ref	Ref
Asian	1 (<1)	0.09 (0.01–0.62)	NA	0	NA	NA	0	NA	NA	2 (1)	0.24 (0.06–0.98)	0.26 (0.06–1.04)
Other	520 (5)	Ref	Ref	469 (4)	Ref	Ref	448 (4)	Ref	Ref	373 (4)	Ref	Ref
Ethnicity												
Non‐Hispanic	443 (5)	Ref	Ref	399 (5)	Ref	Ref	388 (5)	Ref	Ref	325 (4)	Ref	Ref
Hispanic	75 (3)	0.64** (0.50–0.82)	0.59** (0.42–0.81)	65 (3)	0.64** (0.49–0.84)	0.56** (0.39–0.80)	54 (3)	0.55** (0.41–0.73)	0.47** (0.33–0.69)	47 (2)	0.56** (0.41–0.77)	0.54** (0.37–0.79)
SES												
<$100 K	313 (5)	1.37* (1.12–1.67)	1.37* (1.08–1.72)	280 (5)	1.50** (1.22–1.84)	1.63** (1.29–2.04)	226 (4)	1.12 (0.92–1.38)	1.32 (1.04–1.67)	157 (3)	0.86 (0.68–1.07)	1.05 (0.82–1.35)
>$100 K	168 (4)	Ref	Ref	159 (3)	Ref	Ref	189 (4)	Ref	Ref	188 (4)	Ref	Ref

*Note*: Bold indicates significance. *Indicates *p* value ≤ 0.01; ** indicates *p* value ≤ 0.001.

Abbreviations: ADHD, Attention Deficit/Hyperactivity Disorder; aOR, adjusted odds ratio; CBCL, Child Behavioral Checklist; OR, odds ratio; ref, reference; SES, socioeconomic status.

^a^ Percentages are weighted.

^b^ All aOR models were adjusted for sociodemographic factors including race, sex, ethnicity, household income, and parent education as reported in corresponding study wave.

### Patterns of use by race and ethnicity

Throughout the study period, Black/African American (AA) children were almost twice as likely to discontinue ADHD medications compared to White children (9% [*N* = 117] vs. 5% [*N* = 328], OR 1.84, 95% CI 1.45–2.33, *p* < 0.001) (Table 1). Black/AA children were less likely to have continuous medications after 9/10 years of age (2% [*N* = 23] vs. 3% [*N* = 218], OR 0.51, 95% CI 0.33–0.79, *p* < 0.01) and less likely to have continuous use of medications since 9/10 years of age (3% [*N* = 41] vs. 5% [*N* = 315], OR 0.63, 95% CI 0.45–0.91, *p* < 0.01), compared to White children. However, after adjusting for sex, ethnicity, parental education, and household income, differences in patterns of medication usage based on race did not remain significant (Table 1). At baseline and years 1 and 3 of follow‐up Black/AA children were more likely to have elevated CBCL *t*‐scores and not receive ADHD medications compared to White children, however, after adjusting for sociodemographic factors this finding was only statistically significant at baseline (Table 4).

Asian children were significantly more likely to never have received ADHD medications compared to White children (95% [*N* = 183] vs. 87% [*N* = 5577], OR 2.83, 95% CI 1.41–5.68, *p* = 0.004) (Table 1), and there were no statistically significant differences in discontinued or continuous use. This finding remained significant after adjusting for sex, ethnicity, parental education, and household income (Table 1). Asian children had the lowest mean CBCL *t*‐scores throughout the study period compared to all other races (Table 3) and were less likely to have elevated CBCL *t*‐scores compared to children from other races (Table 4).

Throughout the study period Hispanic children were more likely to never have received an ADHD medication (90% [*N* = 1688] vs. 86% [*N* = 6649], OR 1.39, 95% CI 1.17–1.64, *p* < 0.001), and less likely to have continuous use since 9/10 years of age (3% [*N* = 49] vs. 5% [*N* = 373], OR 0. 53, 95% CI 0.39–0.72, *p* < 0.001), compared to non‐Hispanic children (Table 1). This finding remained significant after adjusting for sex, race, parental education, and household income (Table 1). There was no statistically significant difference in discontinuation. As shown in Table 2, Hispanic children were less likely across each wave to receive both stimulant and non‐stimulant medications, compared to non‐Hispanic children. Hispanic children had similar mean CBCL *t*‐scores at each wave compared to non‐Hispanic children (Table 3), and there was no statistically significant difference in elevated CBCL *t*‐scores at each wave (Table 4). However, Hispanic children were less likely to have elevated CBCL *t*‐scores and be taking ADHD medications at each wave of follow‐up (Table 4). This finding remained significant after adjusting for sex, race, parental education, and household income (Table 4).

### Patterns of use by socioeconomic status

As shown in Table 1, children in families with total household income <$100,000 were more likely to discontinue ADHD medications throughout the study period compared to children in families with household income >$100,000. At baseline, children in families with total household income <$50,000 were more likely to receive stimulant and non‐stimulant medications, however, by year 3 they were less likely to receive stimulants and more likely to receive non‐stimulants compared to children in families with income >$100,000 (Table 2).

Throughout the study period, children in families with household income <$100,000 had higher mean CBCL *t*‐scores compared to children in families with income >$100,000 (Table 3). Children in families with income <$100,000 were more likely to have elevated CBCL *t*‐scores and not receive ADHD medications compared to children in families with household income >$100,000 (Table 4). These findings remained significant after adjusting for sex, race, ethnicity, and parental education (Table 4).

## DISCUSSION

In this large national sample of US children, 13% of children had received ADHD medications throughout the study period, consistent with estimates from cross‐sectional national samples (Miech et al., 2023). Findings across 4 waves of data indicate significant disparities in patterns of ADHD medication usage based on sex, race, ethnicity, and household income. Using the longitudinal data of the ABCD Study we found that female children, Asian children, and Hispanic children were more likely to never have used ADHD medications throughout the 4‐year study period. In addition, Black/AA children and children in families with total household income <$100,000 were more likely to discontinue ADHD medications compared to White children and children in families with relatively higher total household incomes.

Findings from this study align with prior findings that female children are less likely to receive ADHD medications compared to male children. Examination of longitudinal data show that, compared to males, females were less likely to have elevated ADHD CBCL *t*‐scores at baseline and first 2 years of follow‐up, however, as time progressed, they were more likely to have elevated ADHD CBCL *t*‐scores compared to males. Furthermore, in later waves, females were more likely to exhibit elevated ADHD symptoms without medication use. This suggests that females with elevated ADHD symptoms are being treated differently in comparison to males with similar symptoms.

Throughout the study period, children in families with total household incomes <$100,000 were more likely to have elevated ADHD CBCL *t*‐scores and less likely to receive ADHD medications, suggesting potential undertreatment in this population. In addition, children in low‐to‐middle‐income families were more likely to discontinue ADHD medications throughout the study period, compared to children in higher‐income families. There are several possible reasons for discontinuation of medications in low‐to‐middle‐income families, including lack or lapse in insurance (Bax et al., 2019; Danielson, Bitsko, et al., 2018; Danielson, Visser, et al., 2018), and inadequate access to health care providers (Hoffmann et al., 2022). Telehealth has been posited to improve this barrier, however, research thus far has not shown that telehealth has eliminated this barrier for disadvantaged populations (Pritchard et al., 2023). Furthermore, the fact that this disparity emerges at middle‐income levels rather than only at low‐income levels suggests it may be attributable to factors specific to high‐income households rather than simply to poverty‐related healthcare disparities. The reasons for this disparity are unclear, prompting questions about differing attitudes toward ADHD medication among affluent families, potentially reflecting distinct social norms or academic priorities compared to low‐ and middle‐income families (Simoni & Drentea, 2016).

Throughout the study period, we found that Black/AA children were more likely to discontinue medications, and also more likely to have elevated ADHD CBCL *t*‐scores and not receive medications at each wave of followup compared to White children. These findings extend prior research by Olfson et al. (2023) in their cross‐sectional study which showed that Black children in the ABCD Study were less likely to receive stimulants at baseline. However, when adjusting for age, sex, ethnicity, household income, and parental education differences in patterns of medication usage between Black/AA children and White children were not statistically significant. This suggests a more complex relationship between race, ADHD symptom severity, and ADHD pharmacotherapy. Specifically, observed disparities were reduced after accounting for sociodemographic variables associated with structural factors such as lower household income and parental education, that may disproportionately impact Black/AA families. Furthermore, while similar rates of elevated ADHD CBCL *t*‐scores were observed between Hispanic and non‐Hispanic children, Hispanic children were less likely to be treated with medications, suggesting potential undertreatment via pharmacotherapy.

Notably, some children may be deprescribed ADHD medications by a health care provider if ADHD symptoms improve and pharmacotherapy is no longer indicated. Indeed, guidelines recommend considering discontinuing medication in patients with sustained periods of remission (Walkup, 2009). In this study, we examined elevated ADHD CBCL *t*‐scores to determine if improving ADHD symptom severity could explain discontinuation. We found that Black/AA children and those in lower‐income families were more likely to have elevated ADHD CBCL *t*‐scores and not receive medications. Similarly, Hispanic children with elevated ADHD CBCL *t*‐scores were also less likely to receive ADHD medications. Therefore, improvement in ADHD symptomatology does not appear to explain disparities in the discontinuation of pharmacotherapy.

Medication adherence in children is largely attributed to parental preference in choice of treatment, that may affect treatment continuity (Fegert et al., 2011; Schatz et al., 2015). Parents in racial and ethnic minority groups may perceive risk versus benefit differently than White parents and may be more concerned about safety and effectiveness of stimulants (Glasofer et al., 2021). Concerns of non‐White parents include the harmful effects of medication therapy and the concern that ADHD medications can lead to substance use disorders (Arcia et al., 2004; Dosreis et al., 2003; Glasofer et al., 2021). Furthermore, as the result of structural racism, Black children and their families have reported mistrust in ADHD treatment as a form of social control and coerced conformance (Glasofer et al., 2021). Clinicians should address culturally based disparities in ADHD treatment by acknowledging implicit bias and promoting shared decision‐making (Glasofer et al., 2021).

### Limitations

There are several limitations to this study. First, although the ABCD Study uses validated scales for assessment of ADHD symptoms, there are no data on clinical diagnosis of ADHD by a health care provider. This study examined ADHD CBCL *t*‐scores which quantifies the severity of parent‐reported internalizing and externalizing behaviors relative to population norms. While the CBCL is not a substitute for a full clinical evaluation, it provides a standardized, widely used measure of ADHD‐related symptoms that reduces bias introduced by clinical diagnoses alone. However, use of parent rating scales alone may impact prevalence of ADHD in the sample as children being actively treated for ADHD may demonstrate lower scores. To overcome this limitation, we examined the combination of CBCL *t*‐scores with medications and without medications, and also used a conservative cut off score of 60 to capture all children with clinically relevant ADHD symptomatology (Biederman, DiSalvo, et al., 2020; Biederman, Fried, et al., 2020; Spencer et al., 2018). Exploring externalizing and internalizing subscales may offer additional insights, particularly in understanding how co‐occurring behavioral problems influence treatment utilization. Second, the ABCD Study is not a nationally representative study. There is significant regional variability in prescribing of stimulants (McDonald & Jalbert, 2013) which may not be captured in the overall study, and limits generalizability of these results. In addition, despite robust efforts to minimize non‐random attrition (Ewing et al., 2018), the ABCD Study sample, when unweighted, is over‐represented by high‐income families (>400% of the federal poverty level [FPL]) and under‐represented by low‐income families (<100% of the FPL or 100%–200% of the FPL). Therefore, survey weights for children in lower‐income families will be larger and more variable than weights for children from higher‐income families (Gard et al., 2023). Third, missing medication data was not randomly distributed and disproportionately affected certain demographic groups. As shown in Appendix 1, participants with missing medication data were more likely to be Black/AA, multiracial, or Hispanic, from lower‐income families, and to have parents with lower educational attainment. Given that these groups already experience disparities in ADHD diagnosis and treatment access, missing data may lead to an underestimation of disparities in medication use. While we conducted complete case analyses, future research should consider multiple imputation or sensitivity analyses to evaluate the potential impact of missing data on findings. Furthermore, some subgroups contained low sample sizes (i.e., Asian children) and therefore should be interpreted with caution. Fifth, our “continuous use” categories were based on parent‐reported ADHD medication use during the 2 weeks prior to each annual study visit, which may not account for treatment gaps or prescription “stimulant holidays” (Martins et al., 2004). Accordingly, our categories should be interpreted as patterns of reported annual use, not uninterrupted daily use. Additionally, as we do not have indications for the medications studied, it may be that medications studied were infrequently used for other indications (e.g., mood). Finally, this study did not assess non‐pharmacological management of ADHD and some children may have received behavioral therapy without ADHD medications. Guidelines for treatment of ADHD recommend a combination of medications and behavioral therapy (Wolraich et al., 2019).

## CONCLUSION

In this longitudinal study of ADHD medication patterns among US children, notable disparities were observed across demographic groups. Female children, Asian children, and Hispanic children were more likely to have never used ADHD medications throughout the study period, while Black/AA children and those from families with household incomes <$10,000 were more likely to discontinue ADHD medications compared to their White and higher‐income peers. The disparities found in female, Black/AA, Hispanic, and low‐ and middle‐income children cannot be entirely explained by differences in parent‐reported ADHD symptoms, suggesting that broader structural factors play a role.

These findings underscore the need for equitable access to health care and ADHD pharmacotherapy for all children with clinically significant symptoms, irrespective of sex, race and ethnicity, or socioeconomic background. Addressing culturally based disparities in ADHD treatment requires a multifaceted approach involving health care providers, policymakers, and communities to ensure equitable diagnosis and treatment of all children with ADHD.

## AUTHOR CONTRIBUTIONS

**Jennie E. Ryan**: Conceptualization; data curation; formal analysis; investigation; methodology; software; writing—original draft; writing—review and editing. **Alexander Weigard**: Conceptualization; data curation; investigation; methodology; writing—original draft; writing—review and editing. **Sean Esteban McCabe**: Conceptualization; methodology; supervision; writing—review and editing. **Timothy E. Wilens**: Conceptualization; methodology; supervision; writing—review and editing. **Philip T. Veliz**: Conceptualization; data curation; formal analysis; investigation; methodology; software; supervision; writing—original draft; writing—review and editing.

## CONFLICT OF INTEREST STATEMENT

The authors declare no conflicts of interest.

## ETHICAL CONSIDERATIONS

Informed consent and assent were obtained from all ABCD Study participants and their parents or legal guardians, as part of the original ABCD Study protocol. Ethical approval for the ABCD Study received centralized approval for data collection through the University of California Institutional Review Board (IRB), and at each individual catchment site. The current study is a secondary data analysis of de‐identified ABCD Study data. This analysis was reviewed and deemed exempt by the Thomas Jefferson University IRB, approval date: February 9th, 2023; IRB reference number: iRISID‐2023‐1411.

## Supporting information

Supporting Information S1

Supporting Information S2

## Data Availability

The data used in this study are publicly available from the NIMH Data Archive (https://nda.nih.gov) as part of the ABCD Study. Access requires an approved Data Use Certification. Results reported in this manuscript are linked to NDA Study 2623 and accessible via https://doi.org/10.15154/gy9e‐0w19.

## APPENDIX 1 SOCIODEMOGRAPHIC COMPARISON OF PARTICIPANTS WITH AVAILABLE MEDICATION DATA VERSUS PARTICIPANTS MISSING MEDICATION DATA

**Table:**

	Participants with available medication data	Participants without available medication data	OR (95% CI)
Total *N* (%)^a^	9708	2157	
Race			
White	6438 (66)	1071 (50)	Reference
Black/African American	1302 (13)	565 (26)	**2.61 (2.32–2.94)**
American Indian/Alaska Native	48 (<1)	15 (<1)	1.92 (1.07–3.44)
Asian	193 (2)	50 (2)	1.56 (1.13–2.14)
Native Hawaiian/Other Pacific Islander	12 (<1)	3 (<1)	1.50 (0.42–5.33)
Other	389 (4)	131 (6)	**2.02 (1.64–2.49)**
Don't know/refuse	102 (1)	45 (2)	**2.65 (1.86–3.79)**
Multiracial	1225 (13)	288 (13)	**1.36 (1.18–1.57)**
Sex assigned at birth			
Male	5090 (52)	1097 (51)	Reference
Female	4616 (48)	1059 (49)	1.06 (0.97–1.17)
Ethnicity			
Non‐Hispanic	7711 (80)	1591 (75)	Reference
Hispanic	1882 (20)	528 (25)	**1.36 (1.22–1.52)**
Household Income^b^			
Below poverty line ($24 k)	1116 (12)	518 (28)	**3.36 (2.93–3.85)**
$25,000–$49,000	1249 (14)	337 (18)	**1.95 (1.68–2.26)**
$50,000–$100,000	2631 (29)	436 (24)	**1.20 (1.05–1.37)**
>$100,000	4005 (45)	554 (30)	Reference
Parent education^b^			
Less than high school	380 (4)	223 (10)	**4.24 (3.54–5.09)**
High school	1021 (11)	420 (20)	**2.97 (2.59–3.41)**
Some college	1509 (16)	437 (20)	**2.09 (1.84–2.38)**
Associate's degree	1236 (13)	297 (14)	**1.74 (1.50–2.01)**
Bachelor's degree or higher	5551 (57)	768 (36)	Reference

*Note*: Bold indicates *p*‐value ≤ 0.001.

Abbreviations: CI, confidence interval; NA, not applicable; OR, odds ratio.

^a^ Percentages are weighted.

^b^ Household income and parental education as reported at baseline.
