# Supplementary material for: Differences in patterns of attention deficit/hyperactivity disorder medication use in US children
Source: JCPP Adv. 2025 Sep 25;6(2):e70040. doi: 10.1002/jcv2.70040 (PMC13260702; doi:10.1002/jcv2.70040)
Supplement: Supplementary file 1 — Supporting Information S1 [file JCV2-6-e70040-s001.docx]

## To classify participants’ reported race in the ABCD we used parent report from the following dataset:

[ABCD Parent Demographics Survey](https://nda.nih.gov/data_structure.html?short_name=pdem02)

Responses were taken from the baseline questionnaire where parents were asked “What race do you consider the child to be? Please check all that apply.” The list of available responses is presented in the table below.

| **Response Option** | **ABCD Variable Name** | **Number of Endorsements** |
| --- | --- | --- |
| White | demo_race_a_p___white | 8796 |
| Black/African American | demo_race_a_p___black | 2517 |
| American Indian, Native American | demo_race_a_p___american_indian | 406 |
| Alaska Native | demo_race_a_p___alaska_native | 5 |
| Native Hawaiian | demo_race_a_p___native_hawaiian | 23 |
| Guamanian | demo_race_a_p___guamanian | 2 |
| Samoan | demo_race_a_p___samoan | 12 |
| Other Pacific Islander | demo_race_a_p___other_pacific_is | 40 |
| Asian Indian | demo_race_a_p___asian_indian | 114 |
| Chinese | demo_race_a_p___chinese | 203 |
| Filipino | demo_race_a_p___filipino | 167 |
| Japanese | demo_race_a_p___japanese | 84 |
| Korean | demo_race_a_p___korean | 100 |
| Vietnamese | demo_race_a_p___vietnamese | 63 |
| Other Asian | demo_race_a_p___other_asian | 90 |
| Other race | demo_race_a_p___other_race | 799 |
| Refuse to answer | demo_race_a_p___refuse_to_answer | 59 |
| Don’t know | demo_race_a_p___dont_know | 104 |

The ABCD Parent Demographics Survey does not offer an option for “multi-racial” but does allow parents to select multiple races. For purposes of analyses, we therefore created a “multi-racial” variable for any parent who endorsed more than one race for the child.

To account for low sample sizes in some racial categories we condensed the following racial categories:

1. American Indian/Alaska Native = American Indian and Alaska Native
2. Asian = Asian Indian, Chinese, Filipino, Japanese, Korean, Vietnamese, or Other Asian
3. Native Hawaiian/Other Pacific Islander = Native Hawaiian, Guamanian, Samoan, or Other Pacific Islander
4. Don’t know/Refuse to answer = Don’t know or refuse to answer

The creation of a “multi-racial” variable and condensing of select racial groups resulted in the following race variable used in all analyses:

| **Race Category** | **Ns** |
| --- | --- |
| White | 7,509 |
| Black/African American | 1,867 |
| American Indian/Alaska native | 62 |
| Asian | 243 |
| Native Hawaiian/Other Pacific Islander | 15 |
| Other | 520 |
| Don't know/refuse | 147 |
| Multiracial | 1,502 |
| Missing | 13 |
