# Supplementary material for: Differences in patterns of attention deficit/hyperactivity disorder medication use in US children
Source: JCPP Adv. 2025 Sep 25;6(2):e70040. doi: 10.1002/jcv2.70040 (PMC13260702; doi:10.1002/jcv2.70040)
Supplement: Supplementary file 2 — Supporting Information S2 [file JCV2-6-e70040-s002.docx]

To classify participants in the ABCD study as “taking ADHD medications” or not, two datasets from ABCD were used:

[ABCD Parent Medications Survey Inventory Modified from PhenX (PMP)](https://nda.nih.gov/data_structure.html?short_name=medsy01)

[ABCD Youth Participant Last Use Survey Day 1 2 3 4 (PLUS)](https://nda.nih.gov/data_structure.html?short_name=abcd_plus01)

Any subject ID/event present in either file was included in the output. In the two datasets, the variable “src_subject_id” corresponds to the subject ID, and “eventname” corresponds to the event.

The possible “eventname” values are baseline_year_1_arm_1, 1_year_follow_up_y_arm_1, 2_year_follow_up_y_arm_1, 3_year_follow_up_y_arm_1, 4_year_follow_up_y_arm_1.

For each subject ID/event, we record whether stimulant or non-stimulant ADHD drugs (or both) are mentioned in either data file. In the medsy01 data file, there are 30 variables searched for ADHD drugs, specifically those with the naming convention: med#_rxnorm_p and med_otc_#_rxnorm_p, where “#” is any integer from 1-15. These are the names of medications taken by the child in the last two weeks, recorded by the parent.

Also, in the medsy01 data file, there are 30 additional variables searched for ADHD drugs, specifically those with the naming convention: med#_rxnorm_1yr_p and med_otc_#_rxnorm_1yr_p, where “#” is any integer from 1-15. These are the names of medications taken by the child in the last year, recorded by the parent. However, the parent also records if the child has taken that medication in the last two weeks. If the child has not taken that medication in the last two weeks, it is removed from the corresponding variable prior to searching.

In the abcd_plus01 data file, there are 22 variables searched for ADHD drugs, specifically those with the naming convention: pls$_med#(_rxnorm_2), where “$” is any integer from 1-4, “#” is any integer from 1-7, and the characters in parentheses may or may not be included. These are the names of medications taken by the child in the last 24 hours, recorded by the child.

According to the documentation linked above, the medication names may include all variants in the [Bioportal RxNorm database](https://bioportal.bioontology.org/ontologies/RXNORM).

A medication was classified as a stimulant ADHD drug if it was included in the variants of any of the ten main ingredients listed under the RxNorm class of “centrally acting sympathomimetics”. The ingredients can be found by searching for that class on the [RxClass website](https://mor.nlm.nih.gov/RxClass/). The list includes amphetamine, armodafinil, dexmethylphenidate, dextroamphetamine, lisdexamfetamine, methamphetamine, methylphenidate, modafinil, serdexmethylphenidate, and solriamfetol. Atomoxetine was removed from the list because it is classified by the FDA as a non-stimulant ADHD drug, as documented [here](https://www.fda.gov/consumers/consumer-updates/treating-and-dealing-adhd).

A medication was classified as a non-stimulant ADHD drug if it was included in the variants of any of the four main ingredients listed by the FDA from the same link above. The list includes atomoxetine, clonidine, guanfacine, and viloxazine.

The variants of each main ingredient were downloaded from the [RxNav website](https://mor.nlm.nih.gov/RxNav/) after searching for that ingredient. Because the site lists generic terms like “Oral Product” and “Pill” in the resulting data file, any record with termType “DF” (Dose Form) or “DFG” (Dose Form Group) was removed. This also means that any record in the ABCD data for which the parent or child selected one of these terms for the child’s medication was removed, as there is insufficient identifying information to classify that medication as being used to treat ADHD or not.

In the data files returned by the RxNav website, there is a code called the RXCUI that identifies the medication. In the ABCD data files, this code is prepended (with a single space) to the medication name. This is the code that is used to identify each medication as being a stimulant ADHD drug, non-stimulant ADHD drug, or neither.

For every medication name listed in the variables of the ABCD data files mentioned above, the RXCUI is extracted, and if there is at least one medication whose RXCUI is contained in the list of RXCUI’s of stimulant ADHD drugs, a flag is set to TRUE for that subject ID/event. The same is done for non-stimulant ADHD drugs.

Since the RxNorm database is updated frequently, and medication names can be added or removed, some medications were missed during the search of the two ABCD data files. To address this, all medication names in either file, whether they are used to treat ADHD or not, were put into a list. Then, for every medication name in that list, a check was performed to see if it contains any of the medication names in our list of ADHD drugs. If the check came back positive, but that medication’s RXCUI wasn’t included in our list of ADHD drugs, it was manually added to the list prior to running the search again.

For example, the RXCUI corresponding to the medication name “clonidine hydrochloride 0.1 MG/ML [Nexiclon]” was initially not in our list of ADHD drugs. However, because the medication name contains the name “clonidine”, which was included in our initial list of ADHD drugs, it was identified through this check. Similarly, the brand name of guanfacine, “Tenex”, was not included in the most recent iteration of the RxNorm database. So, “Tenex” was manually added to the list of medication names to check against. This allowed us to identify two RXCUI’s related to Tenex that were initially missed.

Once confident that all missed medication names of ADHD drugs had been identified, a final search was performed. The output of this search is a data file that contains one row for each subject ID/event, with two identifiers, “adhdStim” and “adhdNonStim”, signifying the presence of stimulant and non-stimulant ADHD drugs in the two original ABCD data files.

All brand names that we searched for are listed below. Other brand names were manually searched for to make sure our list was exhaustive, but if they were absent from both the ABCD data files and RxNorm, they are not mentioned below.

atomoxetine: strattera

clonidine: catapres, clorpres, duraclon, kapvay, nexiclon

guanfacine: intuniv, tenex

viloxazine: qelbree

amphetamine: adderall, adzenys, dyanavel, evekeo, mydayis

armodafinil: nuvigil

dexmethylphenidate: azstarys, focalin

dextroamphetamine: adderall, dexedrine, mydayis, procentra, xelstrym, zenzedi

lisdexamfetamine: vyvanse

methamphetamine: desoxyn

methylphenidate: adhansia, aptensio, concerta, cotempla, daytrana, jornay, metadate, methylin, quillichew, quillivant, relexxii, ritalin

modafinil: provigil

serdexmethylphenidate: azstarys

solriamfetol: sunosi
